# From data collection to design principles: A study of smartwatch faces for enhanced information visualization

**DOI:** 10.1371/journal.pone.0327647

**Published:** 2025-07-02

**Authors:** Miaomiao Dong, Ze Bian, Yuan Zhu

**Affiliations:** 1 College of Art and Archaeology, Hangzhou City University, Hangzhou, China; Takushoku University, JAPAN

## Abstract

With the surge in smartwatch popularity, presenting diverse data on a compact screen poses significant challenges. Previous research has primarily focused on the visual design of smartwatch faces, with limited exploration into how information interacts with users. Moreover, these studies have inadequately addressed interaction and practicality in information visualization. Our work analyzed 518 Huawei and 435 Facer smartwatch faces, synthesizing existing design elements and integrating insights from prior literature to propose a structured design framework that encompasses five key dimensions: content, composition, style, interaction, and practicality. This comprehensive framework not only addresses the visual and functional aspects but also emphasizes the importance of user engagement and individual preferences. By providing detailed guidelines, our framework aims to enhance the interaction, usability, and overall user experience of smartwatch face information visualization, paving the way for more effective and user-centric designs.

## 1. Introduction

In recent years, the rapid advancement and increasing popularity of smartwatches have transformed the way we interact with technology. As these devices become more ubiquitous, the challenge of efficiently presenting useful information on a small watch face has become increasingly significant. The compact size of smartwatch screens demands innovative approaches to information visualization, ensuring that users can quickly and easily access the data they need.

Previous research in this area has made substantial contributions, particularly in motion-based fitness trackers [1], glanceable visualization efficacy [2], real-time feedback [3], heart rate [4,5], sleep [6,7], and diabetes management [8], as well as optimizing graphical and time series data display [9]. However, these studies have primarily focused on the aesthetic and graphical aspects of smartwatch face design, often overlooking the critical interaction between the user and the device. Additionally, there has been a lack of in-depth exploration into personalized design and practicality, which are essential for enhancing user experience and satisfaction.

Most existing studies have relied heavily on data collected from Facer smartwatch faces, a popular platform for custom smartwatch face designs [10,11]. While this data is valuable, it may not fully represent the diverse range of designs available in the market. With the growing popularity of Huawei smartwatches in China, there is a need to broaden the scope of research to include a wider variety of smartwatch faces.

Our work addresses these gaps by conducting a comprehensive study of both Facer and Huawei smartwatch faces. We collected and analyzed 518 Huawei and 435 Facer smartwatch faces, providing a more representative sample of current designs, revising the design framework for information visualization on smartwatch faces, and providing corresponding guidelines for future smartwatch face design. Our main contributions are as follows:

1. Comprehensive Collection and Survey: We conducted an extensive collection and survey of existing Facer and Huawei smartwatch face designs, providing a broad and diverse dataset for analysis.
2. Revised Framework for Information Visualization: By integrating the collected data with a thorough review of relevant literature, we restructured the framework for smartwatch face information visualization. This new framework encompasses five key dimensions: content, composition, style, interaction, and practicality.
3. Comparative Analysis and Design Guidelines: We performed a comparative analysis of the design differences between Facer and Huawei smartwatch faces. Based on our findings and the proposed framework, we provided guidelines to enhance the information visualization on smartwatch faces.

The remainder of this paper is organized as follows: Section 2 reviews related work on information visualization on smartwatch faces. Section 3 presents the extensive collection and investigation of existing Facer and Huawei smartwatch face designs. Section 4 presents the results of the analysis. Section 5 summarizes the design framework for information visualization on smartwatch faces from five aspects. Section 6 presents the discussions and design implications. Finally, Section 7 summarizes our work.

## 2. Related work

With the development of wearable technology, the demand for smartwatches has significantly increased. Displaying diverse data on small smartwatch screens poses significant challenges. Consequently, researchers have dedicated their efforts to exploring various information designs and visualizations tailored to smartwatch displays, as shown in **Table 1**. Considering that the data obtained and displayed by smartwatches are mainly fitness- and health-related information, we divided previous work on information visualization on smartwatches into four types: fitness-related data, health-related data, other data, and multiple data. In addition, we categorize the visualized position of the information, including the smartwatch face, smartwatch application, and techniques, to improve the visualization on smartwatches.

**Table 1:** Recent previous work about information visualizations on the smartwatch.

Information type	Visualized position	Previous work	Specific information type	Research method	Research aspect
Fitness-related data	Smartwatch face	[1]	Sports data	An analysis of 42 sports smartwatch faces	An investigation of the visualization in motion for fitness trackers
		[2]	Calories burned, step count, and active minutes	Three comparison studies with 74 participants	An investigation of the effectiveness of multiple glanceable part-to-whole proportion representations
	Smartwatch application	[3]	Real-time running data	Two user studies with 41 participants	An investigation of the visualization approaches on the smartwatch for real-time feedback
		[13]	Fitness data	A survey with 165 participants; An interview with 20 participants	An exploration of the visualizations to avoid potential negative effects of unmet fitness goals
		[14]	Mountain biking data	A design case study	The design of a crash risk indication application for sports smartwatches
		[10]	Exercise data	The design of 34 smartwatch sketches	An analysis of the results of a full-day context-specific ideation exercise for smartwatch visualizations
		[12]	Activity data	A survey of 80 smartwatch models	An analysis of the user preferred visualization type of activity data
Health-related data	Smartwatch face	[8]	Diabetic health status	A design case study	A design of changing artworks and smartwatch faces to create health status awareness
		[4]	Heart rate data	A questionnaire with 394 participants; A questionnaire with 347 participants	User preferred heart rate data types and visualizations
	Smartwatch application	[15]	Diabetic health status	A design study	A design of the Android-based smartwatch application to monitor the state of diabetes mellitus
		[7]	Sleep data	A survey with 108 participants; One in-person pilot study with 18 participants; Two crowd-sourced studies with 292 participants	An investigation of the preferences and effectiveness of different sleep visualizations
		[20]	In-situ personal health data	A week-long study with 18 participants and a dataset of 205 natural language queries	An investigation of the desired use of a smartwatch for data exploration
		[5]	Heart rate data	A survey with 57 participants; A visualization design; A controlled study with 24 participants	An evaluation of the activity intensity visualizations while playing a Virtual Reality game
Other data	Technique	[16]	Graphic data	Two experimental studies with 23 participants	The design of techniques to improve the visualization on smartwatches
		[17]	Time series data	Two experimental studies with 57 participants	The design of techniques to visualize time series data on smartwatches
		[9]	Inter-related data	Two experimental studies with 25 participants	A design of EdgeSelect, a linear target selection interaction technique that utilizes a small portion of the smartwatch display
Multiple data	Smartwatch face	[18]	Health & Fitness, Weather & Planetary, and Device & Location, other	A survey with 237 participants	An investigation of visualized data type and representation types
		[19]	Personal informatics	A survey with 368 participants; An interview with 18 participants	An investigation of customization needs and preferences in personal tracking
		[11]	Health & Fitness, Weather & Planetary, and Device & Location	An analysis of 358 smartwatch faces	A review and design space on smartwatch faces

For the visualization of fitness-related data, researchers have explored the presentation of different fitness-related data types, such as calories burned, step count, active minutes, real-time running data, and mountain biking data. For smartwatch users, quickly viewing their data during exercise and receiving real-time feedback are beneficial for training. Researchers have investigated the visualization of motion for fitness trackers [1] the effectiveness of glanceable visualizations [2], visualizations for real-time feedback [3], and user preferences for activity data visualization [12]. Additionally, considering the potential negative effects during fitness activities, researchers have conducted studies on visualizations to avoid the negative impacts of unmet fitness goals [13] and the design of visualizations for crash risk indications [14].

For the visualization of health-related data, the focus is on making users more aware of the value of this information and improving the visualization efficiency. Researchers have explored user preferences and visualizations of different kinds of health-related information on the smartwatch face and in the smartwatch application, including heart rate [4,5], sleep [6,7], and diabetic health status [8,15].

Researchers have also explored improvements in design techniques to enhance smartwatch visualizations. Neshati et al. made a series of technical improvements for various types of data on a smartwatch, including the optimization of the display and visualization of graphic data [16], the improvement of time series data visualization [17], and the design of a linear target selection interaction technique to address the issue of insufficient display space on the smartwatch, thereby optimizing the interaction between the user and the device [9].

For the visualization of multiple data, researchers have studied user needs and preferences for information visualizations on the smartwatch face [18,19] and have summarized the design space [11]. Islam et al. conducted a survey of 237 participants to investigate their visualized data and representation types on a smartwatch face. They found that health and fitness data were predominant and that icons accompanied by text was the most frequent representation type [18]. Gouviea et al. conducted a survey of 368 participants and interviewed 18 of them to explore user needs and preferences in personal tracking. They found that users have a need for smartwatch customizations, including data, aesthetic, and personal meaning customizations, but they did not classify these dimensions in detail [19]. Islam et al. analyzed Facer smartwatch face designs, reviewed previous work on information visualizations on smartwatch faces, and summarized the design space, including externals, style/theme, components, and representation [11]. They subdivided these dimensions into specific subcategories, for example, the dimension of representation was subdivided into data types, representation types, and visual features. These studies have provided valuable insights, such as the predominance of health and fitness data, the preference for icon-text combinations, and the desire for customization options. However, they primarily focus on the static visual presentation of smartwatch face designs, with limited consideration given to the interactive aspects between the smartwatch face and the user. While these studies acknowledge the importance of personalization, they do not delve deeply into categorizing and organizing the dimensions of personalization and customization within the context of smartwatch face design. Additionally, the practicality aspect, which is crucial for the real-world application of these designs, has been largely overlooked. Furthermore, the reliance on the Facer platform for data collection introduces certain limitations, as it may not fully represent the diversity of smartwatch face designs available across different platforms and user bases.

To address these gaps, our work aims to provide a more comprehensive analysis and reevaluation of the design dimensions for smartwatch face information visualization.

## 3. An investigation of Facer and Huawei smartwatch face designs

In selecting platforms for our analysis, we prioritized those with significant market presence and influence on design trends in the smartwatch ecosystem. Facer, a widely recognized platform for custom watch faces, was chosen due to its extensive library of over 15,000 unique designs, catering to diverse user preferences and styles (https://www.wareable.com/smartwatches/the-best-android-wear-watch-faces-622). Previous research by Islam et al. utilized Facer as a case study to analyze the diversity and creativity of smartwatch face designs, further validating its role as a key player in this domain [11]. Additionally, Facer’s integration with major smartwatch operating systems, including Wear OS and Tizen, ensures its broad accessibility and adoption across various devices.

Similarly, Huawei was selected as a representative brand due to its dominant position in the global wearable market, particularly in China (https://www.gizguide.com/2024/12/huawei-number-one-wearable-brand-first-three-quarters.html). Recent market reports indicate that Huawei holds a 36% market share for Q3 2024 in China, making it the most widely used smartwatch brand in the region (https://www.huaweicentral.com/huawei-ranks-first-in-china-and-third-in-global-wearable-market-for-q3-2024/). Previous research has also indicated that Huawei is the most widely used smartwatch brand in China [4]. Furthermore, Huawei’s commitment to innovation, exemplified by its advanced health monitoring features and robust design ecosystem, underscores its leadership in shaping industry trend. By analyzing watch faces from both Facer and Huawei, we aim to capture a comprehensive snapshot of the current smartwatch design landscape, ensuring the generalizability of our findings.

We stated that all data were collected from publicly accessible listings on the Facer and Huawei Watch Face Store platforms. The collection and analysis complied with the terms and conditions of both platforms, which permit non-commercial use of publicly available metadata. No private or user-identifiable information was accessed or analyzed.

**Fig 1:**
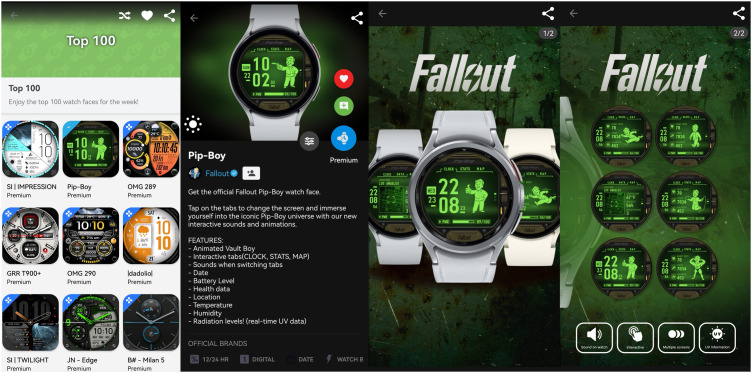
The interface of Facer Top 100 list and the detailed smartwatch face.

**Fig 2:**
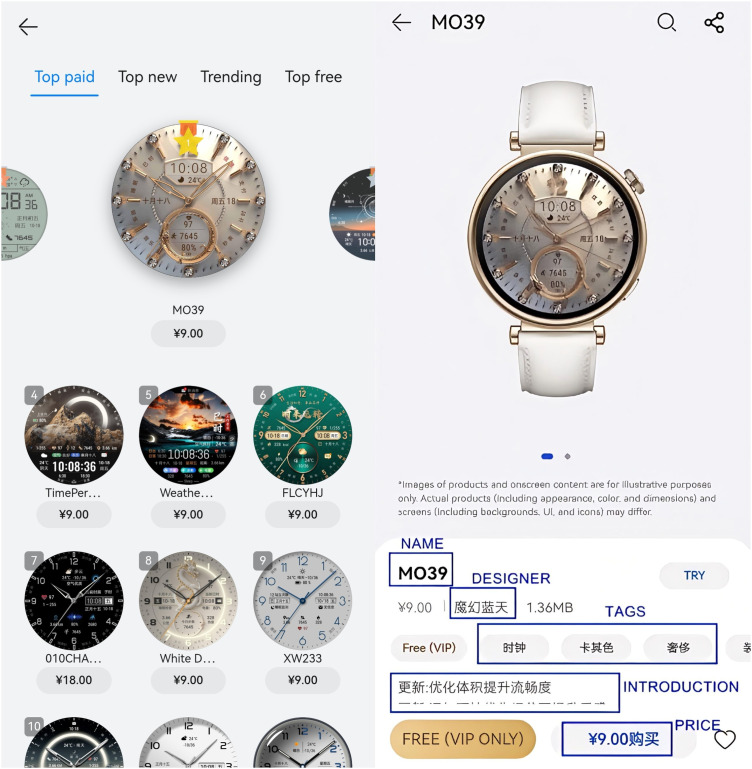
The interface of Huawei Top paid list and the detailed smartwatch face.

The Facer App contains various lists for users, including spotlight, brands you love, new & hot, fun & unique, top 100, etc. The top 100 page lists premium or free smartwatch faces for Apple and WearOS watches, which exhibit a high level of design professionalism. We chose this list for our investigation, as it is more indicative of users’ preferences, as shown in Fig 1. According to a Facer forum, the number of weekly syncs or downloads determines the Top 100 list, which resets every Sunday at midnight (00:00). We manually collected the metadata of the top 100 smartwatch faces every week for about five weeks, from August 8, 2024, to September 15, 2024. The metadata collected for each smartwatch face included the name, introduction, features, and detailed design explanation, as shown in Fig 2. After sorting and removing duplicate smartwatch faces, 435 different smartwatch faces were collected.

The Huawei Watch Face Store contains lists of top-paid, top-new, trending, and top-free. We chose the top-paid list for our investigation, as it is more indicative of Huawei users’ preferences, as shown in Fig 1. According to our observations, this list contained 213 smartwatch faces. It resets every day at midnight (00:00) and changes approximately 10–20 smartwatch faces. We manually collected the metadata of the top-paid smartwatch faces every day at midnight for half a month, from September 10, 2024, to September 25, 2024. The metadata collected for each smartwatch face included the name, introduction, tags, and thumbnails, as shown in Fig 2. After sorting and removing duplicate smartwatch faces, 518 different smartwatch faces were collected.

To conduct a comprehensive comparison and analysis of the 435 Facer faces and 518 Huawei faces, we developed a set of codes specifically tailored to assess the overall design and integration of data representations within the smartwatch faces. These codes, as detailed in Table 2, were designed to focus on the content being represented, as well as the way it was presented. All analyses were conducted using images extracted from each smartwatch interface. We grouped our results according to the codes shown in Table 2. In the dimension of data, we conducted a statistical analysis on the common types of data found in Facer and Huawei smartwatch faces and categorized 20 typical data presentation styles. Regarding the layout, Facer offers both circular and square smartwatch face designs, whereas Huawei primarily focuses on circular ones. For the sake of consistency in our comparative analysis between the two platforms, we only collected and coded the layouts of circular smartwatch faces. In our examination of circular smartwatch face layouts, we paid particular attention to the time display methods and the distribution patterns of information on the dial, which we classified into three main categories: central scattering, up-down arrangement, and left-right arrangement. In terms of other features, based on the data we collected, we focused on five key aspects: primary color, interaction method, power consumption level, memory usage, and price. With regard to interaction methods, we considered whether the smartwatch face supports background switching, shortcuting, dynamic effects, and customization of colors or functions. Additionally, for Huawei smartwatch faces, we extracted and analyzed three extra characteristics—power consumption, memory usage, and pricing—which were not available in the Facer smartwatch faces, possibly due to version differences.

**Table 2:** Codes used in the investigation of Facer and Huawei smartwatch faces.

Dimension	Point	Value
Data	Data type number	1, 2, 3,...
	Steps	Word, icon, number, bar
	Distance	Word, icon, number
	Calories	Word, icon, number, bar
	Active time	Word, icon, number
	Heart rate	Word, icon, number, range
	Sleep	Word, icon, number
	Psychological pressure	Word, icon, number
	Oxygen consumption	Word, icon, number
	Message	Word, icon
	Watch battery	Number, icon, word, bar
	Phone battery	Number, icon, word, bar
	Weather	Word, icon
	Temperature	Number, range
	Altitude	Word, icon, number
	Air pressure	Word, icon, number
	Sunrise & sunset	Word, icon, number
	Moon phase	Word, icon
	Air quality	Word, icon
	Wind speed	Word, icon, number
	Humidity	Word, icon, number
Composition	Time display	Analog, digital, hybrid
	Layout	Central scattering, up-down, left-right
Other features	Color	White, red, black,...
	Interaction	Supporting background switching or not
		Supporting function jump/shortcut or not
		Supporting dynamic effect or not
		Supporting color/function customization or not
	Power consumption level	low level or high level
	Memory	Memory size
	Price	Price number

Three researchers collaborated on coding smartwatch faces. The first author initially coded all faces, which were then reviewed by two additional researchers. Any discrepancies were resolved through group discussion until consensus was reached on all faces. All data and code necessary to replicate this study are available on the OSF Home (https://osf.io/93pzh/).

## 4. Revsults

### 4.1. Data and representation types

#### 4.1.1. Data types.

We conducted an analysis of 435 Facer smartwatch faces and 518 Huawei smartwatch faces, examining the number of data types displayed beyond just the time, and the data types and their quantities are shown in Table 3. Our findings indicated that, on Facer, the average number of data types was 5.06, with a median and mode of 5, and a standard deviation of 2.55, which is similar to the results of previous work that the medians of data type number are 4–5 [11,18]. The range of data types varied from a minimum of 0 to a maximum of 17. In contrast, for Huawei, the average number of data types was 7.63, with both the median and mode at 8, and a smaller standard deviation of 2.17. The range of data types for Huawei also spanned from 0 to 17, which is similar to the results of 16 and 17 in previous studies [11,18].

**Table 3:** Data type and quantity.

Data dimensions	Data types	Platform types and quantities	
Facer	Huawei
Fitness	Steps	370	501
	Distance	116	239
	Calories	63	384
	Active time	0	83
	Total	372	506
Health	Heart rate	315	490
	Sleep	0	189
	Psychological pressure	0	12
	Oxygen consumption	0	6
	Total	315	491
Device	Message	0	421
	Watch Battery	393	484
	Phone Battery	43	0
	Total	393	505
Planetary & Environment	Weather	291	474
	Temperature	351	473
	Altitude	12	115
	Air pressure	0	32
	Sunrise & sunset	46	21
	Moon phase	54	49
	Air quality	0	102
	Wind speed	49	0
	Humidity	40	0
	Total	357	484

These results suggest that Huawei smartwatch faces tend to display a greater variety of information compared to those on Facer. However, due to the physical limitations of the smartwatch face design, the maximum number of data types that can be displayed on a single face, across both platforms, is 17.

When comparing our findings with previous work, we observed that on the Facer platform, the number of watch battery indicators is the highest (393/435, 90.3%), followed by step count (370/435, 85.1%), temperature (351/435, 80.7%), and heart rate (315/435, 72.4%), which is consistent with previous results [11,18]. However, in contrast to the Facer smartwatch faces, the Huawei smartwatch faces show a different distribution: step count is the most frequent (501/518, 96.7%), followed by heart rate (490/518, 94.6%), with watch battery indicators ranking only third (484/518, 93.4%). Additionally, we noticed differences in the presentation of certain data between Facer and Huawei smartwatch faces. For instance, Huawei smartwatch faces do not display data such as phone battery level, wind speed, and humidity, whereas Facer smartwatch faces lack the display of active time, sleep, psychological pressure, oxygen consumption, message, air pressure, and air quality. This indicates a significant difference in the emphasis placed on data presentation between the two platforms. Furthermore, when looking at the broad categories of displayed data, there are also differences in the order of their proportions across the two platforms. On the Facer platform, device-related data has the highest proportion (393/435, 90.3%), followed by fitness (372/435, 85.5%), planetary & environment (357/435, 82.1%), and health (315/435, 72.4%). In contrast, on the Huawei platform, fitness-related data has the highest proportion (506/518, 97.6%), followed by device (505/518, 97.4%), health (491/518, 94.8%), and planetary & environment (484/518, 93.4%).

#### 4.1.2. Representation types of each data.

We analyzed the representation types of each data, calculated the quantity and percentage of each presentation type, and present them in the table below. To more clearly demonstrate the design differences between Facer and Huawei smartwatch faces, we have conducted a comparative analysis of their presentation methods. Due to the differences in the types of data displayed on the two platforms, our comparison and analysis below focus only on the presentation methods of the data that are common to both platforms. For the rest of the specific data, please refer to the Appendix 3 in S1 File. We used the Chi-square test to analyze these categorical data, examining differences between visualization methods and platforms (Huawei and Facer). This test is ideal for categorical variables, which are discrete and non-normally distributed. To ensure validity, we confirmed that each cell’s expected frequency was sufficiently large (≥5) and that observations were independent. These steps ensured the Chi-square test was appropriately applied, allowing us to assess whether observed differences were statistically significant or due to chance.

**Fitness**

**Fig 3:**
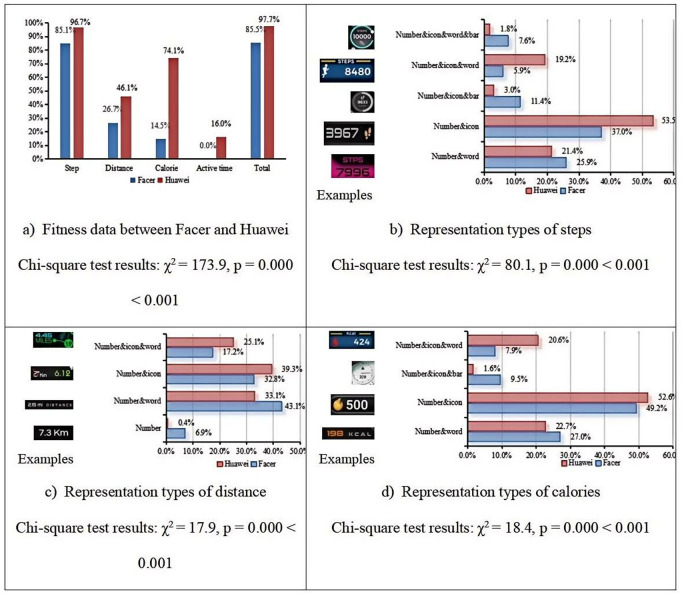
Representation types and comparison of fitness data between Facer and Huawei platform.

We conducted a statistical analysis of the presentation of Fitness data on both the Facer and Huawei platforms, as shown in Fig 3. Fig 3a provides an overall comparison of the data, while Fig 3b, 3c, and 3d illustrate examples and the percentage distribution of step, distance, and calorie data, respectively, across the two platforms. To facilitate a more accurate comparison, we only included data types that are present on both platforms; therefore, the “active time” data, which is not available on the Facer platform, was not included in this analysis. We performed Chi-square tests for these data presented in Fig 3. Our analysis revealed significant differences in the proportions observed for both the Huawei and Facer datasets across different visualization methods (p < 0.05). These results are summarized in Fig 3, which shows the Chi-square test statistics and corresponding p-values for each comparison. As can be seen from Fig 3a, there is a noticeable difference in the proportion of Fitness data displayed on the two platforms, with a higher percentage of fitness data being shown on Huawei smartwatch faces compared to Facer. The disparity is particularly evident for calorie data, where 74.1% of Huawei smartwatch faces display calorie information, whereas only 14.5% of Facer smartwatch faces do so. Furthermore, as illustrated in Fig 3b, 3c, and 3d, for step and calorie data, the “number+icon” display method is the most prevalent on both platforms. However, for distance data, there is a difference: the “number&icon” method is the most common on the Huawei platform, while the “number&word” method is the most frequent on the Facer platform (The inter-group test results of the Chi-square test indicate that there are significant differences).

**Health**

**Fig 4:**
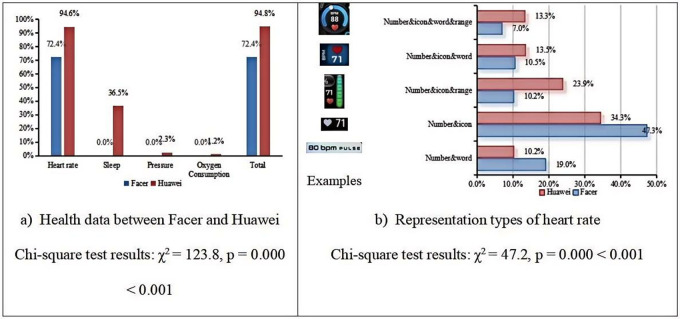
Representation type and comparison of health data between Facer and Huawei platform.

Table 6 presents the statistical overview of health data displayed on Facer and Huawei smartwatch faces. We performed Chi-square tests for these data presented in Fig 4. Our analysis revealed significant differences in the proportions observed for both the Huawei and Facer datasets across different visualization methods (p < 0.05). These results are summarized in Fig 4, which shows the Chi-square test statistics and corresponding p-values for each comparison. As shown in Fig 4a, the Facer smartwatch face only displays heart rate data, lacking other metrics such as sleep, psychological pressure, and oxygen consumption, whereas the Huawei smartwatch face displays a broader range of health data. Fig 4b compares the presentation methods of heart rate data on both platforms, revealing that the “number&icon” format is the most common display type used on both Facer and Huawei smartwatch faces.

**Device**

**Fig 5:**
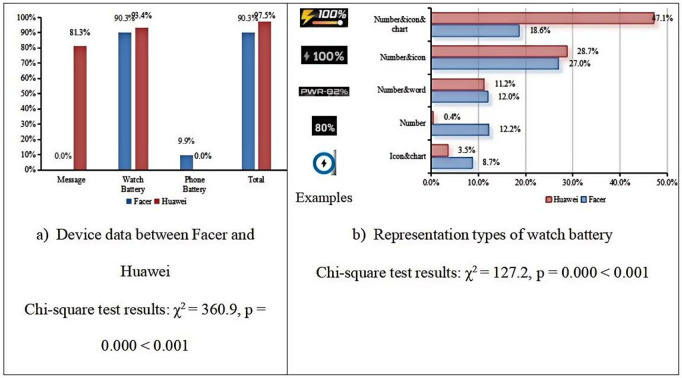
Representation type and comparison of device data between Facer and Huawei platform.

Fig 5 presents the statistical overview of device data displayed on Facer and Huawei smartwatch faces. We performed Chi-square tests for these data presented in Fig 5. Our analysis revealed significant differences in the proportions observed for both the Huawei and Facer datasets across different visualization methods (p < 0.05). These results are summarized in Fig 5, which shows the Chi-square test statistics and corresponding p-values for each comparison. As shown in Fig 5a, both Facer and Huawei smartwatch faces display watch battery levels with a prevalence of over 90%. However, there are significant differences in other types of device data. Message data is more common on Huawei smartwatch faces but does not appear on Facer smartwatch faces. Additionally, Facer smartwatch faces display smartphone battery levels, which are not shown on Huawei smartwatch faces. Fig 5b compares the presentation methods of watch battery data on both platforms, revealing some differences. On Huawei smartwatch faces, the most common display type is “number & icon & chart,” whereas on Facer smartwatch faces, the “number&icon” format is the most prevalent. We also observed that the “number only” display method is used in more than 10% of Facer smartwatch faces, while it is almost non-existent on Huawei smartwatch faces.

**Planetary & environment**

**Fig 6:**
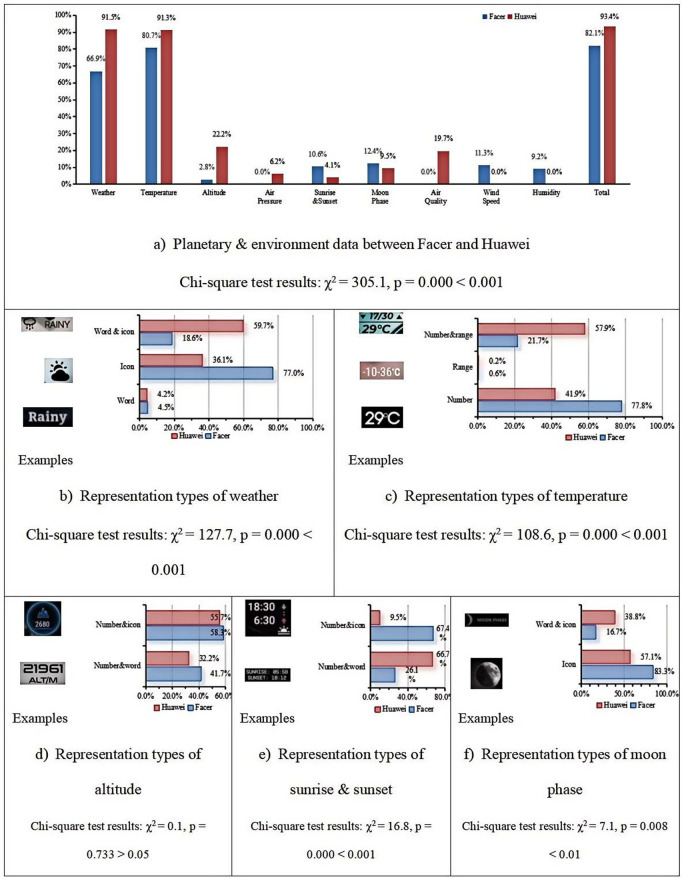
Representation type and comparison of planetary & environment data between Facer and Huawei platform.

Fig 6 presents the statistical overview of planetary and environmental data displayed on Facer and Huawei smartwatch faces. We performed Chi-square tests for these data presented in Fig 6. Our analysis revealed significant differences in the proportions observed for both the Huawei and Facer datasets across different visualization methods (p < 0.05), except altitude. These results are summarized in Fig 6, which shows the Chi-square test statistics and corresponding p-values for each comparison. As shown in Table 6, both Facer and Huawei smartwatch faces have a high prevalence of weather and temperature data. However, there are some differences in other types of data. Facer smartwatch faces display wind speed and humidity data, which are not present on Huawei smartwatch faces. Conversely, Huawei smartwatch faces display barometric pressure and air quality data, which are not available on Facer smartwatch faces. Fig 6b–6f compare the presentation methods of weather, temperature, altitude, sunrise & sunset, and moon phase data on both platforms, respectively. These comparisons reveal certain differences between the two platforms. For instance, in weather data, the “word & icon” format is the most common on Huawei smartwatch faces, whereas the “icon” format is the most common on Facer smartwatch faces. In terms of temperature data, the “number & range” format is the most prevalent on Huawei smartwatch faces, while the “number” format is the most common on Facer smartwatch faces. In the sunrise & sunset data, the “number & icon” format is more common on Facer smartwatch faces, while the “number & word” format is the most prevalent on Huawei smartwatch faces.

These statistical results indicate that, compared to the Huawei platform, Facer tends to favor a more concise data presentation style, while the Huawei platform generally provides a larger amount of information.

### 4.2. Composition

#### 4.2.1. Time display.

**Fig 7:**
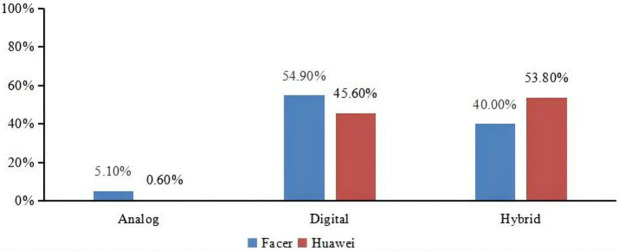
The comparison of time display types between Facer and Huawei platform (Chi-square test results: χ2 **= 31.8, p = 0.000 < 0.001)**.

**Fig 7** shows the time display on Facer and Huawei smartwatch faces. It can be seen that analog types are relatively rare on both platforms. On the Huawei platform, hybrid types predominate, whereas on Facer, digital types are the most common. We performed Chi-square tests for these data presented in Fig 7. Our analysis revealed significant differences in the proportions observed for both the Huawei and Facer datasets across different time display types (p < 0.05). These results are summarized in Fig 7, which shows the Chi-square test statistics and corresponding p-values for each comparison.

For specific numerical comparisons, please refer to the Appendix 4 in S1 File. The following analysis will primarily focus on the comparison between the two platforms, and for the exact numerical values, please refer to the Appendix 4 in S1 File.

#### 4.2.2. Layout.

**Fig 8:**
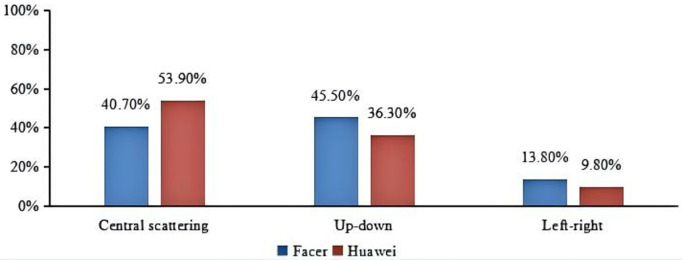
The comparison of layout types between Facer and Huawei platform (Chi-square test results: χ2 **= 16.7, p = 0.000 < 0.001)**.

We conducted a statistical analysis of the smartwatch face layouts, and the results are shown in Fig 8. Central scattering 
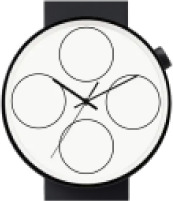
 arranges elements radially; up-down/left-right layouts 
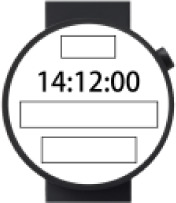
 
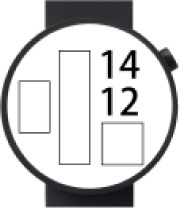
 align components vertically/horizontally. It can be seen that there are some differences in the layouts between Huawei and Facer smartwatch faces. On the Huawei platform, the central scattering type is the most prevalent, with most smartwatch faces mimicking the style of traditional watches. In contrast, on the Facer platform, the up-down type is the most common. This finding is consistent with the results of the time display: Huawei smartwatch faces often feature a mix of analog and digital time displays and tend to replicate the layout of traditional smartwatch faces, while Facer smartwatch faces predominantly use digital time displays, which are typically arranged in an up-down layout. We performed Chi-square tests for these data presented in Fig 8. Our analysis revealed significant differences in the proportions observed for both the Huawei and Facer datasets across different layouts (p < 0.05). These results are summarized in Fig 8, which shows the Chi-square test statistics and corresponding p-values for each comparison.

### 4.3. Other features

#### 4.3.1. Color.

**Fig 9:**
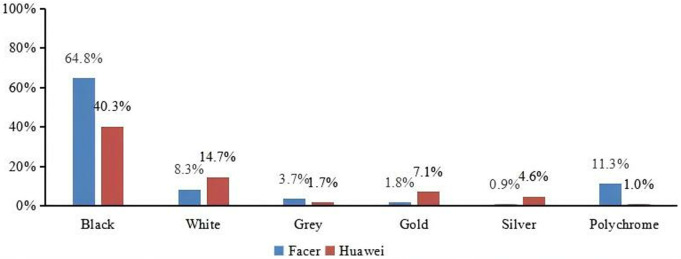
The comparison of main color between Facer and Huawei platform (Chi-square test results: χ2 **= 94.5, p = 0.000 < 0.001)**.

We found that the color of the Huawei and Facer smartwatch faces were not fixed. Many Huawei faces supported a color change, such as time awareness change, that is, displaying white during the day and black at night. Many Facer smartwatch faces supported color customization, allowing users to change the colors of the digits, markers, or partial backgrounds on the smartwatch face. We performed a statistical analysis on the main colors of the smartwatch faces. For Huawei smartwatch faces, if the face color underwent a black-and-white transformation, the main color of the face would be black or white. For Facer smartwatch faces, we extracted and counted the primary colors that remain constant. As shown in Fig 9, the results showed that black was the most common color. In Facer smartwatch faces, aside from black, the most common color is polychrome, followed by white. In Huawei smartwatch faces, aside from black, the most common color is white, followed by gold. We performed Chi-square tests for these data presented in Fig 9. Our analysis revealed significant differences in the proportions observed for both the Huawei and Facer datasets across different colors (p < 0.05). These results are summarized in Fig 9, which shows the Chi-square test statistics and corresponding p-values for each comparison.

#### 4.3.2. Interaction.

**Fig 10:**
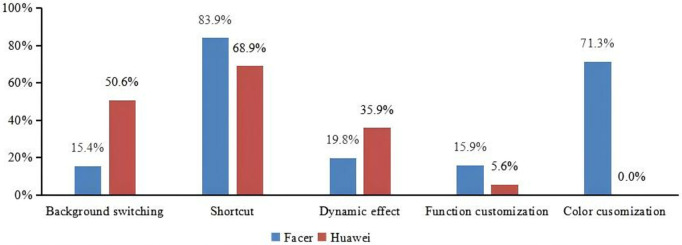
The comparison of interaction between Facer and Huawei platform (Chi-square test results: χ^2 ^ **= 477.1, p = 0.000 < 0.001)**.

We performed Chi-square tests for these data presented in Fig 10. Our analysis revealed significant differences in the proportions observed for both the Huawei and Facer datasets across different interaction methods (p < 0.05). These results are summarized in Fig 10, which shows the Chi-square test statistics and corresponding p-values for each comparison.

**Background switching**

**Fig 11:**
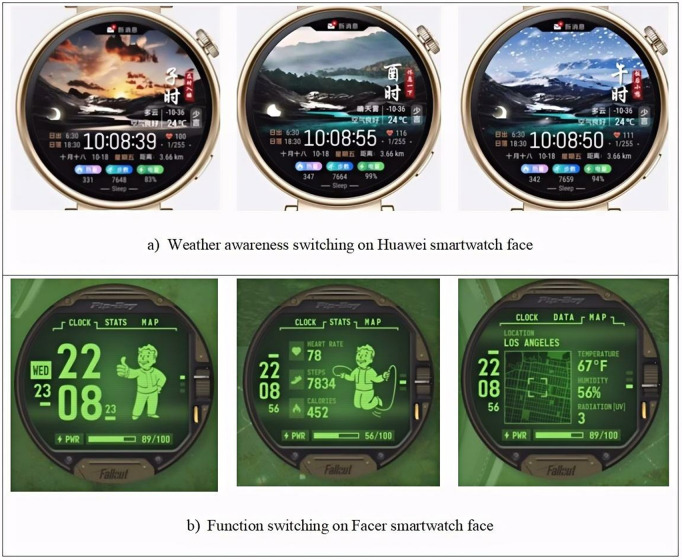
Examples of background switching on Huawei and Facer smartwatch faces.

As shown in Fig 10, according to the statistics, more than 50% of Huawei smartwatch faces support background switching, while only 15.4% of Facer smartwatch faces support this feature. On Huawei smartwatch faces, background switching can include different backgrounds such as colors, weather conditions, mountain landscapes, auspicious characters, and cartoon patterns. The switching modes are also diverse, supporting not only click-based switching but also automatic switching based on time, weather (as shown in Fig 11a), and moon phase, typically without involving the switching of different functionalities. In contrast, on Facer smartwatch faces, background switching is typically click-based, and some smartwatch faces support switching between different functionalities (as shown in Fig 11b). The switching modes on Facer smartwatch faces differ from those on Huawei smartwatch faces.

**Shortcut**

As shown in Fig 10, a majority of smartwatch faces supported shortcuts on both platforms. We define shortcut as not displaying specific data or settings on the smartwatch face but only keeping an area for users to click and jump to another interface to view data or set features. The kept area typically used words or icons to help the users click and jump. The purpose of this function was to save space and facilitate users in selecting functions on the limited smartwatch face.

We analyzed smartwatch faces that supported shortcut, as shown in Table 4. For specific details about the legend content, please refer to the Appendix 1 in S1 File. We found that there are some differences in the shortcut modes between Facer and Huawei smartwatch faces. The majority (249/365, 68.2%) of Facer smartwatch faces do not have word or icon indicators for shortcuts; instead, users can access color or theme picker and adjustments by clicking on blank areas or the center of the smartwatch face. In contrast, many (149/357, 41.7%) Huawei smartwatch faces use word to indicate shortcuts, especially in analog clock faces where word around the dial helps users quickly navigate to the desired functions. Shortcut methods without word or icon indicators are rare on Huawei smartwatch faces, indicating that Huawei’s design is more inclined to provide users with more and clearer information compared to Facer.

**Table 4:** Shortcut details.

Representation type		Word	Icon	Word and icon	No word and icon
Quantity	Huawei	149	105	96	7
	Facer	37	60	19	249

Additionally, we counted the number of shortcuts in the smartwatch faces that support them. Among the 357 Huawei smartwatch faces that support shortcuts, there are a total of 1,978 different types of shortcuts, with an average of 5.5 shortcuts per smartwatch face. Common shortcuts include sleep, payment, and timer functions. In Facer smartwatch faces, among the 365 smartwatch faces that support shortcuts, there are a total of 1,454 different types of shortcuts, with an average of 4.0 shortcuts per smartwatch face. Common shortcuts in Facer include theme pickers and color customization.

**Dynamic effect**

In the statistical analysis of dynamic effects, as shown in Fig 10, we found that more than 30% of Huawei smartwatch faces support dynamic effects. These dynamic effects typically include traditional Chinese elements, such as swimming dragons, koi fish, or the rotation of tourbillons, blooming flowers, and astronaut animations. In contrast, fewer than 20% of Facer smartwatch faces support dynamic effects, and these effects mainly include animations of characters, dynamic background patterns like lines, and other similar visuals.

**Customization**

**Fig 12:**
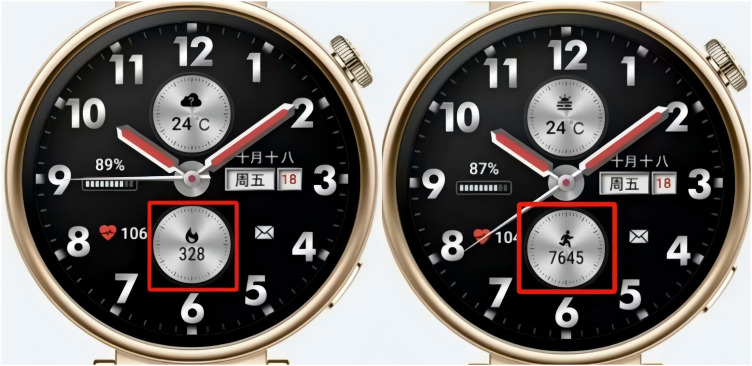
The functional customization of the smartwatch face.

**Fig 13:**
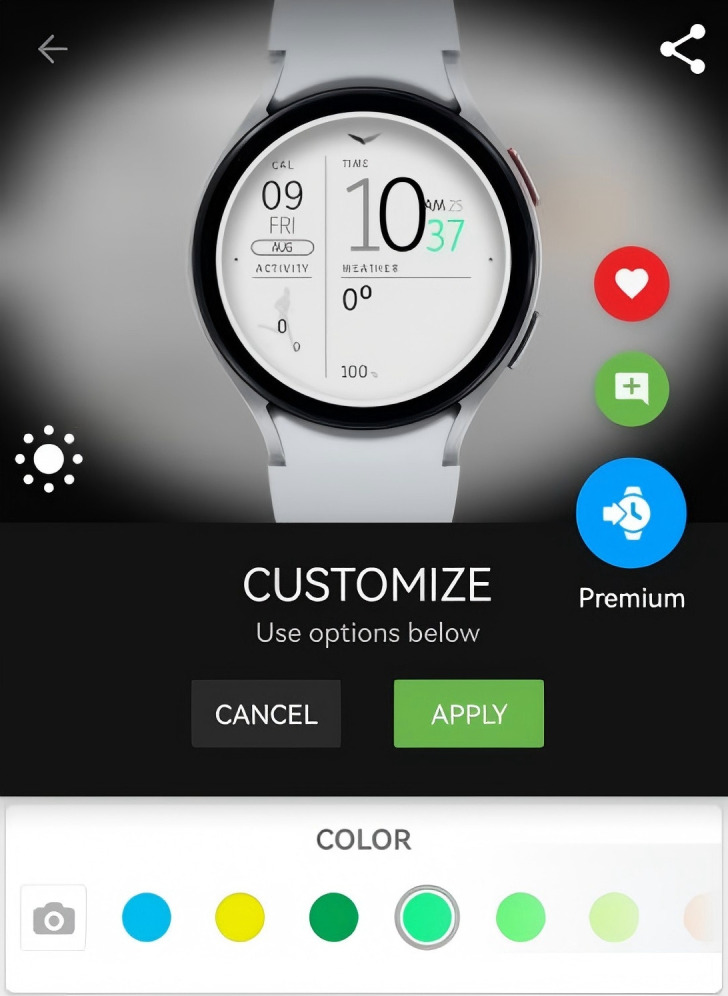
The color customization in the Facer smartwatch face.

Customization can be divided into customization of partial colors within the smartwatch face and customization of certain functions within the smartwatch face (as shown in Fig 12, a specific area on the smartwatch face supports user-defined content display, typically including the display of information such as steps, calories, and heart rate). We have conducted statistics for both types of customization, with the results as shown in Fig 10. It can be observed that only a few Huawei smartwatch faces support functional customization, and Huawei smartwatch faces do not support user customization of the smartwatch face colors; the choice of colors is often reflected in the background switching function, where users can usually switch the background color of the smartwatch face, but the number of options is limited, typically allowing for the replacement of 3–5 colors. In contrast, we found that Facer smartwatch faces offer a greater number of options that support functional customization, and most Facer smartwatch faces allow for the customization of partial smartwatch face colors, as illustrated in Fig 13. Users can choose from over a dozen colors. These results indicate that Facer smartwatch faces provide more customizable features compared to Huawei smartwatch faces, offering users a higher degree of freedom to select the functions and colors they need, with a relatively high proportion of customization.

#### 4.3.3. Power consumption level.

The Huawei smartwatch supports checking the power consumption, which is divided into levels low and high. Level low indicates low power consumption and helps to improve battery life. Level high indicates a 15% reduction in battery life compared to level low. We extracted the degree of power consumption and made a statistical analysis. It was found that 64.7% (335/518) of the smartwatch faces had low power consumption. The remaining smartwatch faces (183/518, 35.3%) were classified as level high and consumed more power.

To explore the factors that influence the power consumption level, we conducted a correlation analysis between the power consumption level and background switching, dynamic effect, and shortcut. Prior to selecting the appropriate correlation method, we tested the normality of the data using the Shapiro-Wilk test (power consumption level: statistic = 0.588, p < 0.001; background switching: statistic = 0.634, p < 0.001; dynamic effect: statistic = 0.595, p < 0.001; shortcut: statistic = 0.563, p < 0.001). The results indicated that the data for all variables did not satisfy the assumption of normal distribution (p < 0.05). Given this, we opted for Spearman’s correlation coefficient analysis, which is more suitable for non-normally distributed data, instead of Pearson’s correlation, which assumes normality. The Spearman’s correlation coefficient analysis results showed that the power consumption level has no correlation with shortcut (Spearman’s correlation coefficient = 0.09, p = 0.07 > 0.05), positively correlated with dynamic effect (Spearman’s correlation coefficient = 0.95, p < 0.001), and negatively correlated with background switching (Spearman’s correlation coefficient = −0.28, p < 0.001).

No information regarding power consumption levels was extracted from the Facer smartwatch faces. Considering that power consumption level is positively correlated with dynamic effects and negatively correlated with background switching, and given that only 19.8% of Facer smartwatch faces support dynamic effects, while 15.4% support background switching—both lower percentages compared to Huawei smartwatch faces—we can infer that the majority of Facer smartwatch faces have low level power consumption. Only a small portion (approximately 10%) of the smartwatch faces have higher power consumption levels.

#### 4.3.4. Memory.

For Huawei smartwatch faces, we extracted the memory of each smartwatch face and performed statistical analysis. The min memory of these smartwatch faces is 0.23 MB, and the max is 4.11 MB. The average memory is 1.60 MB (median: 1.49 MB; mode: 0.85 MB; standard deviation: 0.76).

We conducted a correlation analysis between the memory and power consumption level, background switching, dynamic effect, and shorcut to explore the factors that influence memory. The Spearman’s correlation coefficient analysis results show that memory is positively correlated with power consumption level (Spearman’s correlation coefficient = 0.27, p < 0.001), dynamic effect (Spearman’s correlation coefficient = 0.28, p < 0.001), background switching (Spearman’s correlation coefficient = 0.25, p < 0.001), and no correlation with shortcut (Spearman’s correlation coefficient = −0.06, p = 0.23 > 0.05).

#### 4.3.5. Price.

For Huawei smartwatch faces, we calculated the prices. The average price is 9.3 CNY (approximately 1.3 USD, min: 3 CNY (0.4 USD); max: 88 CNY (12.4 USD); median: 9 CNY (1.3 USD); mode: 9 CNY; standard deviation: 4.6).

The Spearman’s correlation coefficient analysis results showed that price had no correlation with background switching (Spearman’s correlation coefficient = −0.08, p = 0.11 > 0.05). The price had positive correlations with the power consumption level (Spearman’s correlation coefficient = 0.20, p < 0.001), shortcut (Spearman’s correlation coefficient = 0.15, p = 0.003 < 0.01), and dynamic effect (Spearman’s correlation coefficient = 0.18, p < 0.001).

## 5. A design framework

We developed a design framework by reviewing previous studies and existing smartwatch faces from Facer and Huawei platforms to synthesize and integrate the results. This framework enables a thorough analysis of novel visualization features by isolating individual design dimensions for evaluation purposes. We describe these different design choices along five dimensions: content, composition, style, interaction, and practicability, as shown in Table 5. For specific details about the legend content, please refer to the Appendix 2 in S1 File.

**Table 5:** A design framework of information visualization on the smartwatch face.

Dimension	Points	Detail
Content	Data type	Fitness
		Health
		Device
		Weather
		Temperature
		......
	Data type number	1, 2, 3,...
	Representation type	Word
		Icon
		Number
		Chart
		Hybrid
		……
Composition	Device factor	Shape
		Size
		Display resolution
		......
	Time display	Analog
		Digital
		Hybrid
	Layout	Central scattering
		Up-down
		Left-right
Style	Background	Color
		Animation
		Font
	Theme	Texture (Metallic, frosted, smooth,...)
		Topic (Business, luxury, cartoons, sports,...)
		UI style (Flat, sketch, 3D,...)
		......
Interaction	Background switching	
	Shortcut	
	Dynamic effect	
	Customization	Color customization
		Function customization
Practicability	Power consumption	Low High
	Memory	Memory size
	Price	Price number

The identified dimensions were derived from an analysis of the pertinent literature and a review of existing smartwatch faces, employing a bottom-up approach. Three researchers categorized and designated these dimensions. Although the literature and smartwatch face review are not comprehensive, they encompass several seminal and influential studies within the visualization design communities and consider the practical dimension of existing smartwatch faces, which has not been paid attention in the focus of previous research. We believe that these dimensions will serve as a useful framework for categorizing prior and future research on the information visualization on smartwatch faces.

### 5.1. Content

Data and representation are the two most important factors in smartwatch face information visualization. Inspired by previous work on the design space of the dashboard and smartwatch face [11,21], we described the dimension of content as the data contained and its presentation on the smartwatch face, which includes the data type, data type number, and representation type. The data type includes various types of information that appear on the smartwatch face, such as fitness, health, device status, weather, and temperature. Previous work has shown that, although more information is not always better, users want to observe detailed information on the smartwatch face [4]. Therefore, data type number is a key factor to consider. Previous studies have summarized the representation types of information visualization on smartwatch faces, including text, icons, charts, and hybrids [18]. They found that icons accompanied by text were the most frequent types of representations.

Our analysis of smartwatch faces on the Facer and Huawei platforms indicates that the amount of information that can be displayed on such a small screen is limited, with a maximum of 17 types of data. Therefore, it is advisable not to overcrowd the design with too many data types; displaying between 5–8 types is more common. In terms of the types of data displayed, we found that both on Facer and Huawei platforms, the most common types include step count, heart rate, and phone battery level. These data are primarily presented in the form of numbers and icons.

### 5.2. Composition

The primary concern of visualization design is the clear presentation and layout of content on small smartwatch screens. Inspired by previous work [21], we described the dimension of composition as how the time and other data is laid out on the smartwatch face, including the device factor, time display, and layout. The device factor describes the dimensions of the device model, such as shape, size, and resolution. One of the most important functions of the smartwatch is to check time [4], so time display is an essential point in the dimension of composition, which mainly contains three ways to display time: analog, digital, and hybrid [11]. The layout of other data on the smartwatch face can be divided according to the manner of time display, that is, central scattering, up-down, and left-right.

Our analysis indicates that there is a certain correlation between the way time is displayed and the layout. The digital display of time may tend to favor the up-down or left-right layout, whereas an analog or hybrid dial display might lean more towards the central scattering layout. Therefore, these two factors need to be considered in conjunction during the design process.

### 5.3. Style

In previous work, the dimension of style in design has been mentioned [11] In the context of smartwatch face design, style is one of the dimensions that reflects user personalization, aside from content and composition. Referring to the categorization of this dimension from prior studies, we have considered two sub-dimensions: background and theme. The background primarily takes into account the main color of the smartwatch face, font design, and whether animation elements are included. According to our analysis, smartwatch faces with animation elements in the background make up a significant proportion, for both Facer and Huawei smartwatch faces. Common animation elements include cartoon characters, cartoon animals, and elements from anime and games. Some smartwatch faces also include many different types of cartoons or personal characters, such as Maltese, Crayon Shin-chan, and spaceman. Therefore, we have singled out animation elements as a distinct feature. Additionally, when it comes to the theme, we mainly consider the texture, theme topics, and UI-style of the smartwatch face, which all embody the user’s personalized choices. This corresponds to the need for user personalization in smartwatch face design as identified in previous work [21].

### 5.4. Interaction

Previous studies have primarily focused on the dimensional categorization of static smartwatch face designs, with less attention paid to the interaction between users and the smartwatch faces [11]. Based on our analysis of existing Facer and Huawei smartwatch faces, we propose the dimension of interaction, which includes four sub-dimensions: background switching, shortcuts, dynamic effects, and customization. Background switching encompasses both user-initiated clicks to change the background and automatic context-aware switching. For example, the background can change based on time, weather, or moon phase, providing a more diverse interactive experience for users. Shortcuts refer to the fact that, due to the limitations of the small smartwatch face, some information cannot be directly displayed. Instead, icons or words are generally shown, and users can click on them to quickly switch to different functions. Dynamic effects enhance the visual impact by displaying kinetic elements, thereby increasing the sense of experience. Customization includes both color customization and functional customization. Users can choose their preferred colors to change the overall color scheme of the smartwatch face. Additionally, due to the limitations of the smartwatch screen and the varying needs of users, a customization function is provided. This allows users to customize the type of information displayed in certain areas of the smartwatch face. As shown in Table 6, the content within the yellow circle area supports customization, and users can choose to display information such as the number of steps, heart rate, active time, and other data in these areas.

**Table 6:** Mapping key findings to design dimensions.

Design dimensions	Key findings	Analysis
Content	Most common format for data display was a combination of number and icon	Separating number and word treatments could allow for a more refined categorization of functional modules
Composition	Design elements vary across platforms	By comparing designs from Huawei and Facer, we observed differences driven by cultural preferences. For example, time display methods and layout patterns differ significantly between platforms.
Style		
Interaction	Background switching is less commonly applied in Facer smartwatch faces. Functional customization remains limited in both platforms.	Designers need to give more consideration to interaction factors while also being mindful of the balance. This includes implementing power-saving modes or optimizing animations to reduce energy consumption without compromising user experience.
Practicability	Practicability factors, such as power consumption, memory, and price are interrelated with other design elements, which can complicate users’ decision-making processes.	The study revealed that power consumption and memory usage are directly related to interaction complexity. Additionally, pricing influences user preferences; lower-priced watch faces with appealing visual designs often rank higher in paid charts.

### 5.5. Practicability

Prior studies did not consider the dimension of practicability, but it is still a dimension that cannot be ignored and may affect user preferences for smartwatch faces. By reviewing existing Huawei smartwatch faces, we defined this dimension as one that considers practical factors, which mainly concerns three points: power consumption, memory, and price. Smartwatch faces that support dynamic effects may have high power consumption and memory size. In contrast, faces that only support e-ink displays may have low power consumption and a small memory size. In addition, price may also influence users’ preferences for a specific smartwatch face, which should be considered in the visualization design.

## 6. Discussions and design implications

In previous sections, we discussed the importance of incorporating practicability into design considerations. To further enhance the clarity and utility of our proposed five-dimension framework, we have developed Table 6 that maps key findings to each design dimensions. This table aims to provide a clear overview of how specific observations contribute to each dimension, thereby facilitating a better understanding of the relationships between research outcomes and design principles. We also provide design implications for designers to consider when creating smartwatch faces, with the goal of improving and enhancing the user experience by offering more convenient, comprehensive, and personalized smartwatch face designs.

### 6.1. Paying attention to design differences across platforms

Through an analysis of Facer and Huawei smartwatch faces, it becomes evident that there are significant differences in design between the two platforms. Firstly, in terms of the data displayed, although both platforms show common information such as steps, heart rate, weather, and temperature, there are some differences. For instance, Huawei smartwatch faces do not display phone battery level, humidity, or wind speed, whereas Facer smartwatch faces do not show messages, active time, barometric pressure, air quality, or stress levels. These variations may be related to cultural differences among users and the distinct functionalities of the watches. The majority of Huawei smartwatch users are from China, and according to a survey [4], receiving notifications is one of the key reasons for Chinese users to wear a smartwatch; hence, message display is more prevalent on Huawei smartwatch faces, which is not the case with Facer. Additionally, since Huawei watches support user psychological pressure monitoring, this information is featured on their smartwatch faces, unlike on Facer’s.

Moreover, there were notable differences in the compositions of the Huawei and Facer smartwatch faces. In our analysis, 53.8% of the Huawei smartwatch faces displayed the time in a hybrid format, and more than half (53.9%) adopted a central scattering layout resembling traditional mechanical watches. In contrast, 54.9% of Facer smartwatch faces opted for digital time displays and mostly featured up-down layouts. We speculate that this disparity might stem from the difference in smartwatch face shapes. The Huawei smartwatch faces we extracted were exclusively circular, similar to traditional mechanical watches, leading to a preference for needle designs and radial layouts reminiscent of their analog counterparts. Other data information radiates around the needles. However, because of the lower readability of purely mechanical needles, most Huawei smartwatch faces with such designs incorporate digital time displays to enhance their legibility. On the other hand, Facer smartwatch faces come in both circular and square shapes, allowing for more flexibility in design and a stronger inclination toward digital smartwatch face designs, deviating from the conventions of mechanical watches. Additionally, we observed that non-traditional mechanical layouts, such as digital smartwatch faces and up-down layouts, also occupy a considerable proportion of Huawei smartwatch face designs, suggesting that these configurations are prevalent across both platforms.

From an interaction standpoint, Huawei smartwatch faces do not allow for color customization; most can only change the background color or pattern through a switch, with limited functional customization. Conversely, Facer smartwatch faces permit users to customize colors and offer a higher proportion of customizable features, providing greater flexibility to the user.

By comparing these differences, we gain a better understanding of the variances in culture and functionality across different smartwatch brands. Designers should take into account these distinctions and user habits when creating smartwatch faces. For example, when designing for Huawei watches, they might include data on recognized user stress and mood based on the watch’s capabilities. On the other hand, when designing for Facer, a minimalist approach with a focus on a stylish, digital aesthetic could be adopted to meet user needs.

### 6.2. Refining data presentation methods

In terms of information representation, unlike previous studies where ‘text’ encompassed both words and numbers, our work distinguished between text and numbers in the analysis. Our statistical results showed that the most common format for data display was a combination of number and icon (e.g., step count, calories, heart rate, and altitude), aligning with prior research indicating that text and icon is the most frequent form of visualization [18]. Unlike previous work, by distinguishing between numbers and words, we were able to observe more nuanced differences in the data presentation methods on Facer and Huawei platforms. For example, in the display of watch battery levels, we observed that the “number only” display method is used in more than 10% of Facer smartwatch faces, while it is almost non-existent on Huawei smartwatch faces. However, the use of the “number & word” format for displaying battery levels exceeds 10% on Huawei smartwatch faces. If numbers and words were both categorized under “text,” these subtle differences would be difficult to detect. This fine-grained analysis reveals that, compared to the Huawei platform, Facer tends to favor a more concise data presentation style, while the Huawei platform generally provides a larger amount of information. Therefore, separating number and word treatments could allow for a more refined categorization of functional modules, facilitating a more integrated and effective display of information on smartwatch faces.

Both previous research and our study found that despite the limited capacity for data display on smartwatch faces, users prefer to see a variety of information on the faces, which presents significant challenges for both data representation methods and layouts [4]. Given the varying sizes of smartwatch faces and the types of data that they can monitor, it is difficult to establish universal design standards. Designers should strive to refine data representation types, for instance, by distinguishing between word and number within text, using a ‘number+icon’ format for numerical data, ‘word&icon’ for qualitative data, and word for shortcut purposes.

### 6.3. Focusing on the interactive aspects of watch design

In the realm of this design dimension, our work pioneers the inclusion of background switching, dynamic effects, customization, and shortcut within the dimension of interaction, which has not been thoroughly addressed in previous studies. We posit that these attributes underscore the users’ pursuit of individualized, information-rich, and customizable designs, making them indispensable components in enhancing the diversity and richness of smartwatch face designs.

Among these sub-dimensions, background switching, especially context-aware background switching, is less commonly applied in Facer smartwatch faces. Therefore, apart from enriching color palettes, designers can explore background switching mechanisms to enhance visual impact. Current Huawei smartwatch faces implement background changes via click interactions and time/weather/moon phase-awareness, thereby providing valuable examples for designers. They could also consider introducing more diverse triggers, such as activity, heart rate, and mood awareness.

While dynamic effects and shortcuts are relatively common in both Facer and Huawei platforms, the functional customization remains limited. Gouviea et al. highlighted the importance of customized features in smartwatch face design [19]. While users can change backgrounds and select styles, deeper research is needed to customize different functions within pre-existing smartwatch faces. Designers are encouraged to broaden the scope of customization possibilities, allowing users to tailor not just the functions and data types displayed but also the representation methods and display times. This approach grants users greater autonomy, provides them with more choices, enriches the ways and content of interaction, and enhances the overall user experience.

In addition, designers should prioritize dimensions based on user context. Trade-offs are essential and should align with user context and functional priorities. Our analysis underscores the necessity of balancing interactive features with practical constraints. For interaction design, dynamic effects (e.g., animations, rotating elements) significantly enhance user engagement but incur substantial power costs. To mitigate this, designers should prioritize context-aware optimizations: animations could be reserved for luxury or entertainment-focused interfaces, while fitness-oriented faces might employ static progress bars or simplified icons. Shortcuts, though space-efficient, risk visual clutter—hybrid solutions (e.g., subtle icons paired with haptic feedback) can improve discoverability without overcrowding. Customization, while empowering users, demands careful implementation; tiered options (e.g., preset themes for casual users, granular controls for enthusiasts) streamline usability.

### 6.4. Incorporating practicability into design considerations

Prior research has not adequately considered the practicability dimension. In this study, three factors were extracted: power consumption, memory, and price. Our statistical analysis indicates correlations between these practical dimensions and other design aspects. For example, we found a relationship between the dynamic effect and both power consumption and memory; that is, more elaborate dynamic effects generally imply increased energy usage, potentially impacting the battery life of the smartwatch, as well as occupying a larger share of the device’s memory. Additionally, while price did not show strong associations with other design factors, a noteworthy observation from our mean values was that smartwatch faces that entered the top-paid rankings tended to have relatively low prices, with the majority priced at a minimum of 9 CNY (1.2 USD).

Our study is the first to give explicit attention to the dimension of practicability and has revealed how factors within this dimension can influence other design dimensions. Consequently, our findings emphasize the need to consider practicability as a crucial element within the spectrum of design framework when evaluating and creating smartwatch face designs.

Practicability factors, such as power consumption, memory, and price are interrelated with other design elements, which can complicate users’ decision-making processes. For instance, opting for richer and more complex dynamic effects may compromise the memory and battery life. However, we advocate for presenting these practicability factors on the design description pages clearly so that users can make informed choices. Designers should strive to strike a balance between these elements, maintaining dynamic visuals while minimizing battery drain, perhaps by employing idle screen displays to reduce power consumption from animations.

Furthermore, beyond the factors discussed here, designers must consider other practical considerations such as the power consumption associated with idle screen displays. Practicality considerations reveal that power consumption and memory usage are directly tied to interaction complexity. To address this, designers should adopt asset optimization techniques (e.g., vector graphics, compressed files) and integrate user-configurable toggles (e.g., “Power-Saving Mode” disabling animations). Platform-specific adaptations further enhance efficiency: Huawei’s circular screens benefit from centralized layouts to minimize clutter, whereas Facer’s square screens support modular grids for customizable widgets. Transparency in design metrics (e.g., labeling “High Power” for animated faces) empowers users to make informed trade-offs. By aligning interaction richness with technical feasibility, designers can deliver engaging yet resource-efficient smartwatch experiences.

### 6.5. Limitations

Our study has several limitations that should be acknowledged to provide a balanced view of our findings.

First, the smartwatch face list collected from Facer includes both premium and free smartwatch faces, with data captured between August 8th, 2024, and September 15th, 2024, while the list from Huawei consists solely of paid smartwatch faces, captured between September 10th, 2024, and September 25th, 2024. These discrepancies in data collection periods and types of smartwatch faces may introduce sample selection bias. The data collection period for Facer spans five weeks, while the Huawei dataset covers only half a month, which could result in variations in user preferences and trends over time due to seasonal or promotional factors. Additionally, the inclusion of both premium and free smartwatch faces in the Facer dataset allows for a broader range of design practices and user preferences to be analyzed, whereas the exclusive focus on paid smartwatch faces in the Huawei dataset may skew results towards more professionally designed and visually sophisticated options favored by users willing to pay for them. Given that Huawei has a significant market presence, particularly in China, the paid smartwatch faces in the Huawei dataset may reflect the preferences of a more affluent user base, while the Facer dataset captures a wider diversity of user preferences across different demographics and regions. In addition, although Facer and Huawei are highly representative platforms within their respective domains, they may not fully encapsulate the entirety of the smartwatch ecosystem.

These differences impact the generalizability of our findings. Specifically, when comparing the visualization methods and design elements between Facer and Huawei datasets, it is important to consider the potential influence of these biases, as the higher prevalence of professionally designed faces in the Huawei dataset may lead to an overestimation of the sophistication of design elements. While our Chi-square tests indicate significant differences in proportions across various visualization methods (p < 0.05), these results should be interpreted with caution given the potential biases introduced by the sampling differences. While generalizability is limited to these platforms, our comparative analysis highlights culturally driven design preferences. To mitigate these biases, future studies should aim to collect data over consistent time frames and include a balanced mix of premium and free smartwatch faces across platforms. Incorporating user feedback and demographic information could provide further insights into the preferences and behaviors of different user groups.

Second, although we endeavored to encapsulate a wide array of factors within the design framework pertinent to smartwatch faces, certain aspects of smartwatch face design may have been inadvertently overlooked. For example, prior studies did not consider the dimension of practicability, but it is still a dimension that cannot be ignored and may affect user preferences for smartwatch faces. Therefore, summative conclusions regarding the design framework may not be exhaustive, hinting at potential areas for further exploration in future research. Future work should address these gaps by expanding the design framework to include additional dimensions like practicability and conducting more comprehensive analyses to ensure a holistic understanding of smartwatch face design and user preferences.

## 7. Conclusions and future work

In conclusion, while previous research has primarily concentrated on the visual aesthetics of smartwatch faces, it has fallen short in addressing the interactive and personalized aspects of information visualization. Our study, which analyzed 518 Huawei and 435 Facer smartwatch faces, addresses these gaps by proposing a comprehensive design framework that encompasses five key dimensions: content, composition, style, interaction, and practicality. This framework not only enhances the visual and functional elements but also emphasizes user engagement and individual preferences, thereby enriching the overall user experience.

Our findings highlight the importance of several design implications. First, designers should pay attention to the differences across platforms, tailoring their designs to the unique features and user bases of each. Second, refining data presentation methods is crucial for effectively conveying information on the compact screen of a smartwatch. Third, focusing on the interactive aspects of watch design, such as context-aware background switching and dynamic effects, can significantly enhance user engagement. Finally, incorporating practicability into design considerations ensures that the smartwatch faces are not only visually appealing but also functional and user-friendly. By providing detailed guidelines, our framework aims to improve the interaction, usability, and overall user experience of smartwatch face information visualization, setting a new standard for more effective and user-centric designs.

The focus on pre-existing watch faces from these platforms may overlook emerging trends driven by smaller or niche brands that contribute to the diversity of smartwatch designs. Future research could expand the scope of analysis to include other platforms and brands, as well as explore user-generated content beyond the curated selections offered by Facer and Huawei. In the future, we will delve deeper into information visualization on smartwatch faces, extending the statistical analysis to cover smartwatch faces from other design platforms to further refine and optimize the design framework. Moreover, while the current study establishes a robust foundation for our proposed design framework through comprehensive reviews and platform analyses, future research will validate this framework via extensive user surveys and expert reviews. We will gather feedback from a diverse group of users across various demographics and engage with industry experts to refine the framework, ensuring it addresses user expectations and the complexities of smartwatch design. Integrating these perspectives aims to enhance the framework’s practical credibility and broad applicability.

## Supporting information

S1 File **Appendix 1.** Specific details for Table 4. **Appendix 2.** Specific details for Table 5. **Appendix 3.** Presentation types and quantity of each data types. **Appendix 4.** Different dimensions and quantity of Facer and Huawei platform.
(DOCX)
