# Supplementary material for: From data collection to design principles: A study of smartwatch faces for enhanced information visualization
Source: PLoS One. 2025 Jul 2;20(7):e0327647. doi: 10.1371/journal.pone.0327647 (PMC12221094; doi:10.1371/journal.pone.0327647)
Supplement: S1 File — Appendix 1. Specific details for Table 4. Appendix 2. Specific details for Table 5. Appendix 3. Presentation types and quantity of each data types. Appendix 4. Different dimensions and quantity of Facer and Huawei platform. (DOCX) [file pone.0327647.s001.docx]

Appendix

1. **Specific details for Table 4**

1. **Specific details for Table 5**

1. **Table 7. Presentation types and quantity of each data types**

| Data dimensions | Data types | Presentation types | Quantity | |
| --- | --- | --- | --- | --- |
|  |  |  | Facer | Huawei |
| Fitness | Steps | Icon&bar | 4 | 2 |
|  |  | Word&bar | 1 | 1 |
|  |  | Number&word&bar | 18 | 3 |
|  |  | Number&word | 96 | 107 |
|  |  | Number&icon | 137 | 268 |
|  |  | Number&icon&bar | 42 | 15 |
|  |  | Number&icon&word | 22 | 96 |
|  |  | Number&icon&word&bar | 28 | 9 |
|  |  | Bar | 2 | 0 |
|  |  | Number | 17 | 0 |
|  |  | Number&bar | 3 | 0 |
|  | Distance | Number | 8 | 1 |
|  |  | Number&word | 50 | 79 |
|  |  | Number&icon | 38 | 94 |
|  |  | Number&icon&word | 20 | 60 |
|  |  | Word&icon | 0 | 4 |
|  | Calories | Icon&bar | 1 | 3 |
|  |  | Number&word | 17 | 87 |
|  |  | Number&icon | 31 | 202 |
|  |  | Number&icon&bar | 6 | 6 |
|  |  | Number&icon&word | 5 | 79 |
|  |  | Number&word&icon&bar | 1 | 7 |
|  |  | Number&word&bar | 2 | 0 |
|  | Active time | Number&word | 0 | 31 |
|  |  | Number&icon | 0 | 38 |
|  |  | Number&word&icon | 0 | 14 |
| Health | Heart rate | Icon | 0 | 1 |
|  |  | Icon&range | 5 | 1 |
|  |  | Number&range | 0 | 1 |
|  |  | Number&word | 60 | 50 |
|  |  | Number&icon | 149 | 168 |
|  |  | Number&icon&range | 32 | 117 |
|  |  | Number&icon&word | 33 | 66 |
|  |  | Number&icon&word&range | 22 | 65 |
|  |  | Range | 1 | 0 |
|  |  | Number | 3 | 0 |
|  |  | Word&icon | 2 | 0 |
|  |  | Icon&word&range | 1 | 0 |
|  |  | Number&word&range | 7 | 21 |
|  | Sleep | Word | 0 | 56 |
|  |  | Icon | 0 | 51 |
|  |  | Number&word | 0 | 5 |
|  |  | Icon&word | 0 | 63 |
|  |  | Number&icon | 0 | 12 |
|  |  | Number&word&icon | 0 | 2 |
|  | Psychological pressure | Number&word | 0 | 2 |
|  |  | Number&icon | 0 | 3 |
|  |  | Number&word&icon | 0 | 6 |
|  |  | Word | 0 | 1 |
|  | Oxygen consumption | Number&word | 0 | 3 |
|  |  | Number&icon | 0 | 1 |
|  |  | Number&word&icon | 0 | 2 |
| Device | Message | Word | 0 | 11 |
|  |  | Icon | 0 | 302 |
|  |  | Word & icon | 0 | 108 |
|  | Watch Battery | Chart | 7 | 3 |
|  |  | Icon&chart | 34 | 17 |
|  |  | Number | 48 | 2 |
|  |  | Number&word | 47 | 54 |
|  |  | Number&icon | 106 | 139 |
|  |  | Number&icon&chart | 73 | 228 |
|  |  | Number&icon&word&chart | 29 | 13 |
|  |  | Word&chart | 13 | 0 |
|  |  | Icon | 1 | 0 |
|  |  | Icon&word&chart | 3 | 0 |
|  |  | Number&word&chart | 18 | 0 |
|  |  | Number&icon&word | 6 | 16 |
|  |  | Number&chart | 8 | 12 |
|  | Phone Battery | Number&icon | 4 | 0 |
|  |  | Word&chart | 2 | 0 |
|  |  | Number&word | 16 | 0 |
|  |  | Number&icon&chart | 8 | 0 |
|  |  | Number&word&chart | 4 | 0 |
|  |  | Icon&chart | 6 | 0 |
|  |  | Number&icon&word&chart | 1 | 0 |
|  |  | Number&word&icon | 2 | 0 |
| Planetary & Environment | Weather | Word | 13 | 20 |
|  |  | Icon | 224 | 171 |
|  |  | Word & icon | 54 | 283 |
|  | Temperature | Number | 273 | 198 |
|  |  | Range | 2 | 1 |
|  |  | Number&range | 76 | 274 |
|  | Altitude | Number&word | 5 | 37 |
|  |  | Number&icon | 7 | 64 |
|  |  | Number&word&icon | 0 | 14 |
|  | Air pressure | Number&word | 0 | 15 |
|  |  | Number&icon | 0 | 12 |
|  |  | Number&word&icon | 0 | 5 |
|  | Sunrise & sunset | Number&word | 12 | 14 |
|  |  | Number&icon | 31 | 2 |
|  |  | Number&word&icon | 0 | 5 |
|  |  | Number | 3 | 0 |
|  | Moon phase | Word | 0 | 2 |
|  |  | Icon | 45 | 28 |
|  |  | Word & icon | 9 | 19 |
|  | Air quality | Word | 0 | 85 |
|  |  | Number&icon | 0 | 2 |
|  |  | Word&icon | 0 | 14 |
|  |  | Number&word&icon | 0 | 1 |
|  | Wind speed | Number&word | 25 | 0 |
|  |  | Number&icon | 9 | 0 |
|  |  | Number&word&icon | 15 | 0 |
|  | Humidity | Word | 1 | 0 |
|  |  | Number | 5 | 0 |
|  |  | Number&word | 15 | 0 |
|  |  | Number&icon | 19 | 0 |

1. **Table 8. Different dimensions and quantity of Facer and Huawei platform**

| Dimensions | Dimension types | Quantity | |
| --- | --- | --- | --- |
|  |  | Facer | Huawei |
| Time display types | Analog | 22 | 3 |
|  | Digital | 239 | 236 |
|  | Hybrid | 174 | 279 |
| Layout types | Central scattering | 177 | 279 |
|  | Up-down | 198 | 188 |
|  | Left-right | 60 | 51 |
| Color | Black | 282 | 209 |
|  | White | 36 | 76 |
|  | Grey | 16 | 9 |
|  | Gold | 8 | 37 |
|  | Silver | 4 | 24 |
|  | Polychrome | 49 | 5 |
| Interaction | Background switching | 67 | 262 |
|  | Shortcut | 365 | 357 |
|  | Dynamic effect | 86 | 186 |
|  | Function customization | 69 | 29 |
|  | Color cusomization | 310 | 0 |
